# Stress-Adaptive Stiffening Structures Inspired by Diatoms: A Parametric Solution for Lightweight Surfaces

**DOI:** 10.3390/biomimetics9010046

**Published:** 2024-01-12

**Authors:** Selina K. Linnemann, Lars Friedrichs, Nils M. Niebuhr

**Affiliations:** Alfred Wegener Institute Helmholtz Centre for Polar and Marine Research, Am Handelshafen 12, 27570 Bremerhaven, Germany; selinakayla@gmail.com (S.K.L.);

**Keywords:** combs, diatom frustules, lightweight design, parametric design, surface stiffening, stress adaptation

## Abstract

The intricate and highly complex morphologies of diatom frustules have long captured the attention of biomimetic researchers, initiating innovation in engineering solutions. This study investigates the potential of diatom-inspired surface stiffeners to determine whether the introduced innovative strategy is a viable alternative for addressing engineering challenges demanding enhanced stiffness. This interdisciplinary study focuses on the computer-aided generation of stress-adaptive lightweight structures aimed at optimizing bending stiffness. Through a comprehensive microscopical analysis, morphological characteristics of diatom frustules were identified and abstracted to be applied to a reference model using computer-aided methods and simulated to analyze their mechanical behavior under load-bearing conditions. Afterwards, the models are compared against a conventional engineering approach. The most promising biomimetic approach is successfully automated, extending its applicability to non-planar surfaces and diverse boundary conditions. It yields notable improvement in bending stiffness, which manifests in a decrease of displacement by approximately 93% in comparison to the reference model with an equivalent total mass. Nonetheless, for the specific load case considered, the engineering approach yields the least displacement. Although certain applications may favor conventional methods, the presented approach holds promise for scenarios subjected to varying stresses, necessitating lightweight and robust solutions.

## 1. Introduction

Diatoms have long been a source of inspiration in biomimetic engineering, primarily owing to their unique silicified frustules that showcase remarkable properties and intricate patterns [1,2]. The frustules fulfill a multitude of crucial functions, with protection being one of the most significant ones [3,4,5]. As highly abundant primary producers, diatoms are extensively targeted by a range of organisms [6]. Pathogens, parasitoids, and predators use diverse chemical and mechanical strategies to access the cell content, involving herbivores such as copepods, nematodes, gastropods, and Euphausiacea, each functioning at different size scales [7]. Consequently, there is a substantial demand for their frustules to be highly resistant and offer efficient protection [8]. However, particularly in the case of pelagic diatoms, the imperative to stay in the upper water column for photosynthesis poses a challenge. Thus, the frustule must offer protection while maintaining a lightweight nature. Researchers assume that the diverse patterns and structures observed in diatom morphology stem from the array of feeding strategies to which diatoms are subjected [9]. This inherent combination of properties makes them particularly intriguing for use in technological applications, such as lightweight engineering and architecture [2], where engineers and scientists seek solutions to minimize weight while preserving mechanical performance.

In recent decades, advances in computational and microscopic methods have enabled scientists to study the mechanical properties of diatoms more thoroughly (e.g., [4,9,10,11,12]). Computer-aided approaches, such as the combination of parametric design and simulation, can be a useful tool to complement theoretical and experimental approaches to better understand the functional morphology of diatom frustules.

Hamm et al. analyzed the strength of several centric and pennate diatoms, revealing that the frustules were able to withstand forces equivalent to a maximum of 700 t/m^2^ [4]. Additionally, they were able to use simulations to show that removing the ornamented structures and adding a simple thickening of the frustule with the same amount of material led to more than 70% higher von Mises stress and displacement values compared with the unaltered ribbed frustule models. Zglobicka and Kurzydlowski used different microscopic methods to create simulations of a computer-based model of parts of the frustule under bearing [13]. They concluded that the ribbing structures significantly improve the resistance against localized forces. It was also proven in several studies that the architecture of the frustule leads to a heterogeneous stress distribution when force is applied [4,9]. Costae (ribs) smoothly deflected stress peaks by absorbing stress from more vulnerable areas, such as the areolae. Gutiérrez et al. performed simulations to analyze the unit cell of *Coscinodiscus* sp. with varying dimensions of their geometrical features [14]. Three-point bending simulations reveal that the honeycomb sandwich structure contributes to the high strength observed in *Coscinodiscus* sp. This is an important structural attribute, as local stress hotspots in a structure increase the risk of failure.

Studies have been conducted to create bio-inspired lightweight structures. For example, Maier et al. employed a method to use a suitable natural archetype, such as radiolarians, to tackle a corresponding technical challenge, employing it as a design space to create adapted lightweight design solutions [15]. Breish et al. presented two novel diatom-inspired approaches to optimize the stiffness of plates and cellular solids by employing a gradient material distribution strategy [16]. The authors were able to produce models with maximum displacement values close to topology optimization, even outperforming it in some cases. Parametric design has been investigated as an option to create biomimetic solutions in lightweight engineering [17,18,19]. As a subcategory of computer-aided design (CAD), parametric design is a method in which the parameters or variables that define the shape, size, and behavior of a model are specified mathematically or algorithmically [20]. These characteristics may be modified and altered while relationships are maintained. This allows the investigation of various design possibilities and setups, with changes inherited automatically throughout the model [21]. Most of the studies implementing parametric design focus on the development of lattice structures, with a focus on the development of variable designs (e.g., [18]).

Even though these recent studies explore diatom-inspired adapted lightweight structures, a noticeable gap persists in the development of automated adaptive stiffening solutions derived from diatom-inspired principles. In particular, the potential of computational design methods in this matter, such as parametric design, has yet to be fully realized. This research aims to bridge this gap by introducing a novel approach that combines biomimetic inspiration with advanced parametric design to create efficient and adaptable stiffening structures for surfaces.

We present a comprehensive investigation into the development of a stress-adaptive lightweight stiffening structure inspired by diatom frustules. It is assumed that diatom-inspired lightweight structures can offer more efficient surface stiffening than conventional engineering methods. The primary objective was to implement and compare different approaches to create diatom-inspired stress-adaptive surface stiffeners and to examine their potential in comparison to a conventional method in lightweight engineering. By exploring the potential of diatom-inspired stiffening structures, this study seeks to contribute to the development of sustainable and efficient engineering solutions. By combining the insights from diatom-inspired biomimetics with the power of computational design, we aimed to contribute a novel solution to the scarcity of parametric adaptive stiffening structures.

## 2. Materials and Methods

### 2.1. Experimental Approach

**Figure 1:**
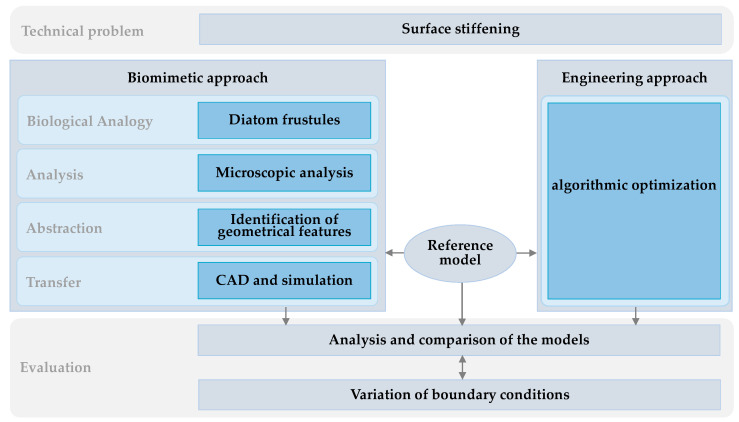
Overview of the approach undertaken in the present study.

Two main approaches were used to minimize the displacement of a plate (Figure 1). The biomimetic approach comprised several steps. Firstly, a biological analogy was identified for the given technical problem, which in this study was represented by diatom frustules. Frustules of different genera were then microscopically analyzed to examine their morphological characteristics. These geometric features were abstracted and applied to a reference model using computer-aided methods. The reference model consisted of a computational model of a planar plate with defined load cases and boundary conditions. The second approach was algorithmic optimization applied to the reference model to compare the biomimetic model with a conventional approach in mechanical engineering. The resulting models were evaluated in terms of their maximum overall displacement. Subsequently, the framework conditions were varied to investigate the robustness of the established methodology.

### 2.2. Reference Model

The models were designed parametrically using the low code-based software Synera (version 23.05, Synera GmbH, Bremen, Germany). The mechanical behavior of each constructed model was determined using the solver Optistruct (Altair^®^ HyperWorks^®^ Version 2019, Troy, MI, USA), which is implemented in the Synera interface, and was integrated into the parametric workflow.

**Figure 2:**
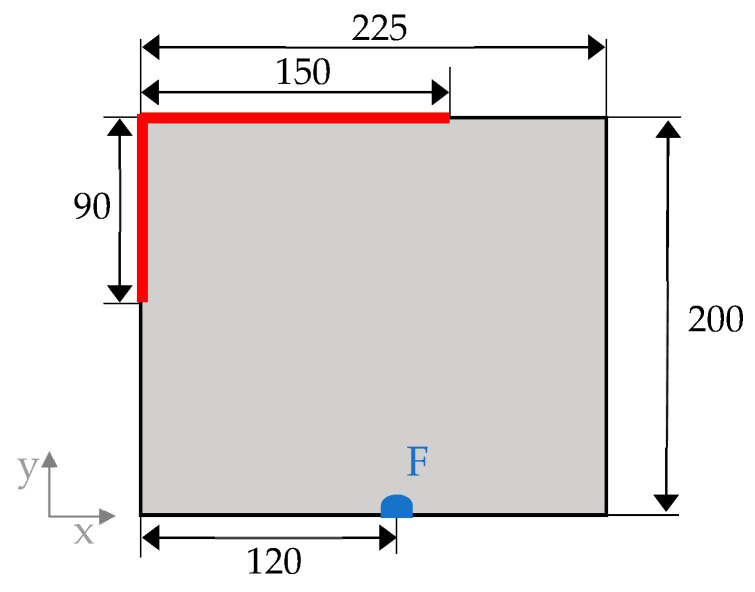
Sketch of the reference model with its dimensions. The red line indicates the fixed support, and the blue semi-circle depicts the area where F was applied in a positive z-direction.

A reference model was set up as a planar rectangular plate with the dimensions shown in Figure 2**.** The thickness t of the plate was set to t=2.78 mm to result in a total mass $m_{t}$ of 1 kg. The model was constrained in all degrees of freedom along the red lines. A static load F=1 kN was applied to the plate in the area of the blue semi-circle with a radius of 5 mm in the positive z-direction. In the next step, a shell mesh was generated for the model. The meshing was performed using the Optistruct mesher, which is implemented in the Synera software. To achieve a sufficient element density for the model, a mesh study was carried out, varying the average element size $l_{e}$. For more information, please refer to Pegg et al. [22]. Structural steel is assigned as the material for all models presented in this study, with the properties specified in Table 1. In principle, the choice of material for isotropic structural investigations is of minor importance, provided that identical materials are employed for both the developed and reference models. In industrial applications, the choice of material depends on the specific application and production requirements. To analyze the influence of the structural aspects of the models, the material properties and the mass were set as constants for all models.

Afterward, the reference model was numerically solved using the finite element method to analyze its stiffness. For more information, please refer to Hartmann and Katz [23]. In this study, the focus was set on bending stiffness $K_{B}$. This quantifiable measure refers to the ability of a material or structure to resist deformation or bending when subjected to a load or an applied force [24]. According to Hooke’s law, stiffness can be defined as the ratio of the applied force F to the resulting displacement $\delta_{max}$, and it depends on the material and the structure’s shape and dimension.

(1) $$K_{B}=\frac{F}{\delta_{max}}$$

Another measure directly related to stiffness is the second moment of area I, which is a geometrical property used to calculate the deflection of a beam caused by a moment or force applied to the beam [24]. I depends on the geometry of the cross-section, where the distance to the neutral axis plays a crucial role. Bending stiffness is influenced by Young’s modulus E multiplied by I, leading to the following relationship:

(2) $$K_{B}=E\times I$$

As the models were subjected to the same load case and had the same material properties, the relative stiffness was analyzed solely by comparing the maximum displacement $\delta_{max}$ of the models. The von Mises stress $\sigma_{VM}\;$distribution in the reference model resulting from this load case served as the basis for some of the presented models.

### 2.3. Engineering Approach: Thickness Optimization

To analyze and compare the performance of the biomimetic approach accordingly, thickness optimization was applied to the reference model to represent a conventional engineering approach. For the optimization, the “Optistruct Optimizer” implemented in Synera was used. The 2D elements were initially assigned a uniform thickness according to the reference model. The possible element thickness was constrained to be between 1 mm and 15 mm. A design constraint was set to match $m_{t}=1\;kg$. The optimization objective of the algorithm was defined as minimizing $\delta_{max}$. The optimized model typically results in an uneven surface, which should ideally be remodeled into a smooth 3D mesh. As the other models were constructed using 2D meshes, this model was also not further converted to maintain comparability.

### 2.4. Biomimetic Approach: Stress-Adaptive Sandwich Combs

#### 2.4.1. Microscopical Analysis of Diatoms

The diatom frustules were analyzed using Scanning Electron Microscopy (SEM) and Confocal Laser Scanning Microscopy (CLSM). The samples for the analysis were provided by the Alfred Wegener Institute (AWI) and originated from their collection of diatom cultures. Some cultures contained only one species, which was isolated from a sample and propagated. Other cultures contained a mix of genera. The study area was not provided. The diatom samples were stained using the in vivo stain Lysosensor DND-160 (PDMPO, Fischer Scientific GmbH, Schwerte, Germany) according to the protocol by Desclés et al. [25]. The diatoms were grown in an f/2 medium [26] and dosed with Lysosensor DND-160 at a final concentration of 125 µM. For the freshwater diatoms, the WC medium by UTEX was used.

To remove the organic contents of the cells and the frustulin membrane coating the frustule, the diatoms were washed in deionized water four times by centrifuging at 453× *g* and subsequently removing the supernatant. The pellet was then dosed with a saturated KMnO_4_ solution and incubated for 24 h at ambient temperature. This solution was carefully mixed with an equal volume of concentrated HCl and boiled until the mixture cleared. Five washing steps to remove the chemicals, as described above, followed. The cleaned frustules were dried on microscopic slides and mounted in Prolong Antifade Glass mountant (Fischer Scientific GmbH, Schwerte, Germany). For SEM, the cleaned frustules were air-dried on a round 10 mm cover slide and mounted on SEM stubs using double-sided conducting carbon tabs. To ensure good conductivity, the samples were sputter coated with a 20 nm gold/palladium layer. The samples for light microscopy were cleaned and mounted in Naphrax (Biologie-Bedarf Thorns, Deggendorf, Germany), a synthetic resin for diatom analysis with a very low refractive index of 1.71.

SEM images were captured using a FEI Quanta 200 FEG SEM (FEI, Hillsboro, OR, USA). SEM was set to 10 kV accelerating voltage, and the images were captured using secondary electron (SE) imaging. The CLSM images were obtained using an Olympus Fluoview FV10i confocal laser scanning microscope with an excitation wavelength of 405 nm and emission wavelength of 540 nm. The images were acquired using a 60× oil immersion objective with a numerical aperture of 1.435. The image stacks were acquired at an XY resolution of 1024 × 1024 pixels and 200 nm for the z-planes. The resulting image stack was rendered into a 3D model using Bitplane Imaris 7.3.

The last step of the microscopic analysis was the examination of the images to examine geometrical features. For the unidentified diatoms, taxonomic keys were used to identify genera based on characteristic features of the frustule. The morphological features within the different genera do not differ significantly for our purposes, and identification at the species level is not necessary for this study. The structural elements were then evaluated in terms of their properties and their significance for mechanical performance. By understanding the purpose of the frustule’s structural characteristics, knowledge of the form and function of the frustules was gained to abstract and transfer their principles. The focus of this study lies on the structural properties; therefore, the material properties of the frustules were neglected.

#### 2.4.2. Comb Studies

The abstracted morphological features of the diatom frustules were transferred to the plate stepwise to analyze the influence of their characteristics on the displacement of the model. A uniform thickness was assigned to the plate of t=1 mm and t=1.2 mm for any added ribbing or sandwich structures. All following patterns were created as curves, which were then extruded in the positive z-direction to create a ribbing structure. The height of the ribbing structures was uniform and calculated to match the restriction of $m_{t}=1\;kg$. The resulting models were analyzed with regard to their maximum displacement under the application of the previously defined load case.

**Figure 3:**
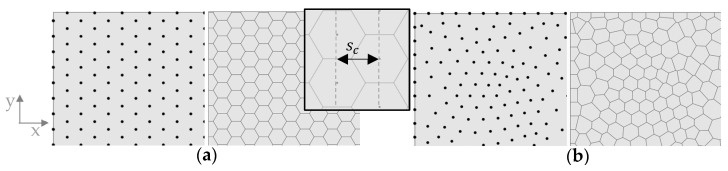
Voronoi combs are based on (**a**) left: a point grid based on mathematical relationships; right: a resulting uniform hexagonal pattern after application of the Voronoi diagram and a definition of the comb size $s_{c}$. (**b**) left: a randomized 2D point distribution; right: a resulting irregular polygon field after application of the Voronoi diagram.

The basis for the different comb patterns in this study was a Voronoi diagram, which is a mathematical method used to divide a space into regions based on proximity to specified points [27]. In a two-dimensional space, it is generated by computing the perpendicular bisectors of the line segments between each pair of points and intersecting these bisectors to form the edges of the Voronoi polygons. First, a 2D point distribution is generated on the surface, which will control the shape and distribution of the combs (Figure 3).

A first study was conducted to analyze the influence of the comb size $s_{c}$ and rib height $h_{c}$ on $\delta_{max}$ for a model with regular hexagonal combs. A regular point field was created manually to result in uniform hexagons based on a mathematical distribution, considering that all interior angles of a hexagon are 120 degrees each (Figure 3a). To analyze the size, $s_{c}$ was gradually increased from 5 mm to 25 mm in 2 mm steps. Here, the comb size is meant as the distance in the x-direction of the center points of two rows of hexagons, as indicated in Figure 3.

Subsequently, the influence of the uniformity of the combs was tested. Two different point distributions were used to apply a Voronoi diagram to compare a model with a regular hexagonal pattern and a model with an irregular polygonal pattern. The regular model was chosen from the previously performed size study, with a favorable $s_{c}$ based on the maximum overall displacement. For the second model, a random point distribution was created to generate an irregular pattern of different polygons (Figure 3b). This point distribution is controlled by defining a global distance value $d_{c}$, which defines the average distance between points. This value was set according to the value $s_{c}$ for the regular pattern.

As a next step, stress adaptive patterns were introduced. For this approach, the density of the combs was varied based on the von Mises stress distribution in the reference plate resulting from the applied load case. This means that areas with higher stress values had a higher density of combs, resulting in smaller comb sizes, while areas with lower stress values had a lower density of combs. For this reason, the calculated stress distribution of the reference model was translated into a stress field SF. To achieve this, the centers of the finite elements and the stress response in said points were extracted. The field then consisted of points with individual stress values, the SF was further used to manipulate the comb patterns. The thickness of the elements, the height of the ribs, and the mass of the model were constrained in the same way as in the previous models.

Three different patterns were aimed for. The first one, pattern A, consisted of irregular polygons with adapted densities. Similar to Figure 3b, a random point distribution was used; however, instead of one global distance value $d_{c}$ to control the point distribution, the previously defined stress field was used in this workflow to manipulate the density locally. Subsequently, the stress values of the field were normalized and then remapped to values between 8 and 30, which served as local distance factors. Afterwards, the Voronoi combs were applied. For patterns B and C, the regular pattern illustrated in Figure 3a served as a base for the adapted pattern. For both, the feature “transform morph” was used, which distorts a geometry based on a spatial field of local scaling factors. As a first step, the values of the SF were remapped to values between 0 and 3. Afterwards, the values were additionally evaluated as x for the mathematic expression as follows:

F(x) = 1/(1 + exp(−((x − 0.7) × 10)))(3)

By applying this expression, the values were changed from a linear distribution to an S-shaped distribution, resulting in a higher contrast between the low and high values. These values served as the scaling factors $s_{f}$ from 0 to 1 so that the maximum stress value equals $s_{f}=0$ and the minimum stress values equal $s_{f}=1$, which means no distortion. These values were then set as local fields and combined into one field. The resulting field of scaling factors thus distorted the hexagonal pattern. For pattern C, the same method was applied, but instead of distorting the curves of the hexagonal pattern, the center points were morphed, and the Voronoi field was applied after the distortion.

**Figure 4:**
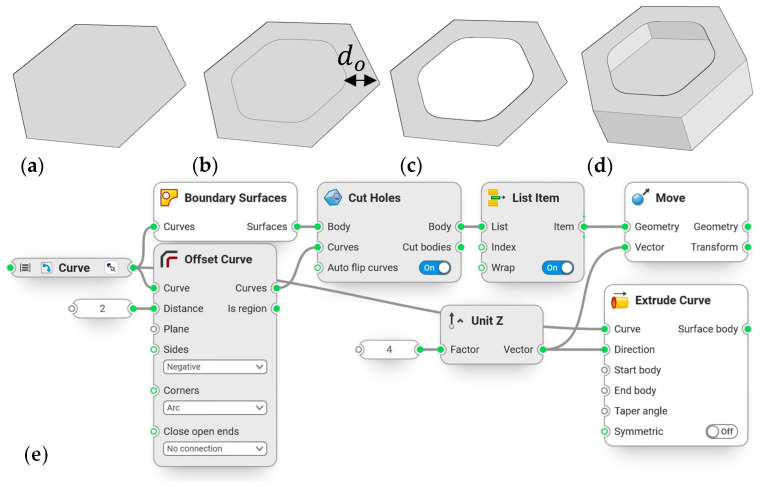
Simplified process of creating a hexagonal comb with a sandwich structure. (**a**) Create a surface based on a boundary curve; (**b**) offset the boundary curve with offset distance $d_{o}$; (**c**) obtain the resulting surface after cutting out the inner surface; (**d**) move that surface up, extrude the boundary curve, and combine with the boundary surface; (**e**) combine with the associated workflow in Synera. The outputs of the white features are the ones that make up the final geometry.

For the last part of the comb studies, a new feature, defined as a sandwich structure, was introduced. A surface was added on top of the combs to generate a structure that resembled the foramen found in many diatom genera (Figure 4c). The sandwich structure was generated for the model with the lowest $\delta_{max}$, chosen in the previous section. Figure 4 shows the steps to create this structure for a simplified example of a single hexagonal comb, which was created for the whole model. For this study, different offset distances $d_{o}$ from 0.5 mm to 1.5 mm were applied, and the maximum overall displacement and rib heights were compared.

#### 2.4.3. Combined Model

The findings obtained from the individual steps conducted in the preceding chapter were integrated synergistically to create a novel model. This chapter included the creation of two models, both based on the pattern with the lowest maximum displacement obtained from the sub-chapter on adaptive patterns. In contrast to previous models, a variable rib height was employed. This rib height was tailored to match the stress field of the reference model and ranged between 0 and 15 mm. Furthermore, a sandwich structure was introduced but applied only to regions exhibiting high-stress values $\sigma_{VM}$. The stress field of the reference model was divided into distinct regions according to the percentages outlined in Table 2. These intervals were chosen intuitively, as the resulting geometry effectively portrays the different areas by representing the division of the stress field.

No supplementary stiffening structures were incorporated in regions where stress values remained below 10% of the maximum stress threshold of 600 MPa. Conversely, for regions with stress values exceeding 10%, comb structures were introduced. This was achieved by initially creating a non-planar assistance surface, which enabled the adjustment of rib heights for the combs. The stress field values were translated into a comb height range spanning from 1 to 15 mm, where 0 MPa corresponded to $h_{c}=1\;\mathrm{mm}$ height, and 600 MPa corresponded to $h_{c}=15\;mm$.

The first step involved generating a planar point grid on the reference plate, followed by the generation of vectors to move these points. By evaluating the stress field, which was translated into heights, at each point and multiplying the apparent value with the z-unit vector, the height was assigned as the amplitude of the vector. Subsequently, the moved points served as a foundation for creating the non-planar assistance surface. This assistance surface was then used to intersect the extruded combs, resulting in a varying rib height, adjusted based on the stress distribution. The addition of sandwich structures involved filtering out combs with stress values ranging from 40% to 100%. Only these selected combs were used to create an offset, according to Figure 4. The offset distance was chosen according to the results from the sub-chapter on sandwich structures. As a last step, thickness optimization was applied to the stiffening structures. The base plate was set to 1 mm and was not part of the optimization process. The possible thickness was set between 0.5 to 3 mm. The objective was to minimize the maximum overall displacement, and a defined constraint was a maximum total mass of $m_{t}=1\;kg$.

The second model was an improvement of the first model in this chapter. By analyzing the results of the thickness optimization, areas of the combs assigned a small thickness were reduced in area by cutting out an oval shape. This reduction of material was used to increase the maximum rib height to 20 mm. As in the model before, a thickness optimization was run with the same constraint and objective, but for this model, the thickness of the plate was included in the optimization as well, with a possible thickness between 0.5 and 1.5 mm.

### 2.5. Variation of Boundary Conditions

To analyze the robustness of the created workflow, the applicability of the combined approach for changing boundary conditions was explored. Thus, a randomly designed non-planar surface was constructed, and a new load case was defined. Two approaches were tested in addition to the new reference model: thickness optimization on the bare surface and the improved combined model.

**Figure 5:**
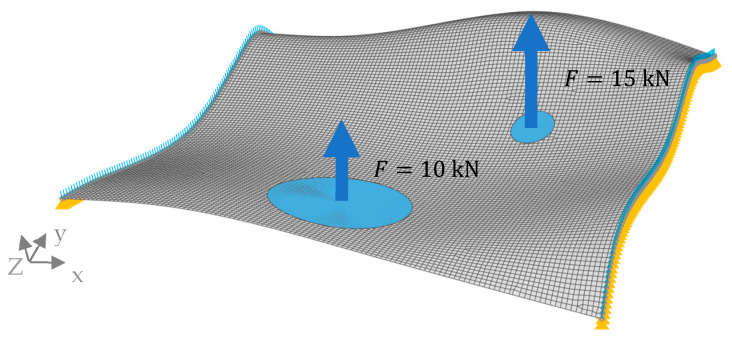
Non-planar surface and the applied configuration. The yellow triangles on the two opposing edges of the surface indicate the pinned support restricted in the translatory z-direction. The blue circles indicate the load application in the positive z-direction.

For the new load case, the surface was supported by a pinned support configuration, with movement restricted solely in the translatory z-direction. This restriction was applied on two opposing edges of the surface, illustrated by the yellow-highlighted edges in Figure 5. Additionally, two static loads were applied to circular areas on the surface, as indicated in the figure.

For the non-planar reference model, the thickness was adjusted to ensure $m_{t}=1\;kg$, as in the previous models. The thickness optimization was run under the same conditions as previously defined for the planar model. The parametric workflow for the improved combined model was adjusted to easily switch between planar and non-planar surfaces. The initial conditions were modified to input the new surface design and configuration. The subsequent step involved evaluating the surface’s behavior by analyzing the maximum overall displacement of each model and verifying its suitability.

## 3. Results

### 3.1. Reference Model

**Figure 6:**
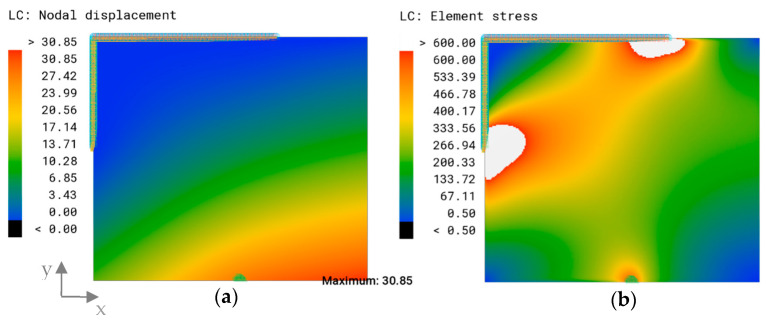
(**a**) Overall displacement plot and maximum overall displacement (mm) and (**b**) von Mises stress distribution (MPa) of the reference model under load case 1. The maximum stress threshold was set to 600 MPa.

The model was considered adequately connected for $l_{e}=2\;mm$. However, since the optimized models involved more complex geometries, and a uniform mesh size is preferable, the mesh size $l_{e}=1\;mm$ was chosen for meshing all models. The displacement plot of the reference model showed an increasing displacement towards the lower right corner, where the maximum overall displacement was assessed as $\delta_{max}=30.85\;mm$ (Figure 6a).

The related von Mises stress distribution showed stress hot spots in single elements. As the stress distribution was not an objective of this study but a reference for the patterns, the maximum threshold was changed so that all values higher than 600 MPa, which are depicted using white in Figure 6b, were set to 600 MPa to attain a smooth stress field.

### 3.2. Engineering Approach: Thickness Optimization

**Figure 7:**
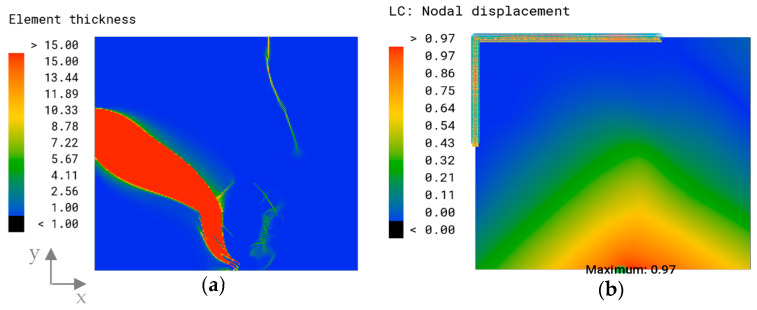
(**a**) Element thickness plot (mm) and (**b**) overall displacement plot and maximum overall displacement (mm) of the optimized model.

Figure 7a shows the thickness plot of the algorithmically optimized model for the objective of a minimized overall displacement. Most of the elements were assigned the minimal element thickness of 1 mm, indicated in blue. Discrete rib-like areas with a maximum thickness of 15 mm (red color) and a transitioning zone with a medium thickness (green) value were formed. The thicker area expands from the left edge of the fixed support to the area of force application. The maximum overall displacement of this model was $\delta_{max}=0.97\;mm$ and appears in the area of load bearing (Figure 7b).

### 3.3. Biomimetic Approach: Stress-Adaptive Sandwich Combs

#### 3.3.1. Microscopic Analysis of Diatoms

**Figure 8:**
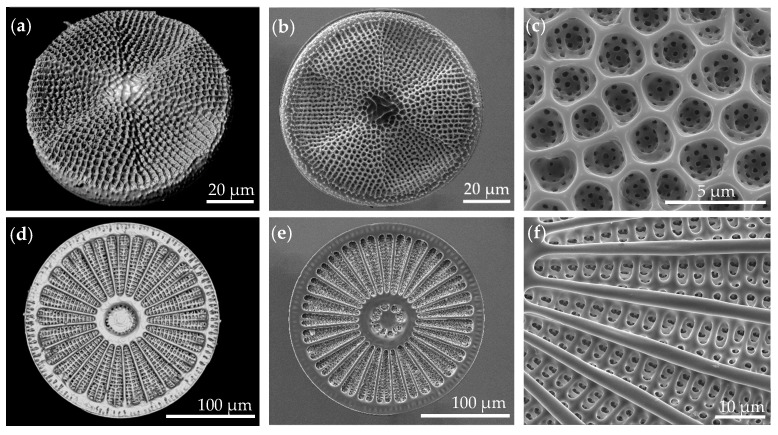
Upper row: microscopy images of *Actinoptychus* sp. Lower row: microscopy images of *Arachnoidiscus* sp.. The images (**a**,**d**) were taken using CLSM, and (**b**,**c**,**e**,**f**) were taken using SEM.

A total of 17 genera were identified using the microscopic methods. The frustules exhibited a high morphological diversity, yet common geometries were observed. The focus of this study was set on combs and their characteristics. Figure 8 shows exemplary images of frustules exhibiting comb structures for both microscopic methods. The 3D models generated using CLSM allowed for the analysis of structures from different perspectives and provided a more accurate impression of the structures, while the images from SEM provided a high degree of detail.

**Figure 9:**
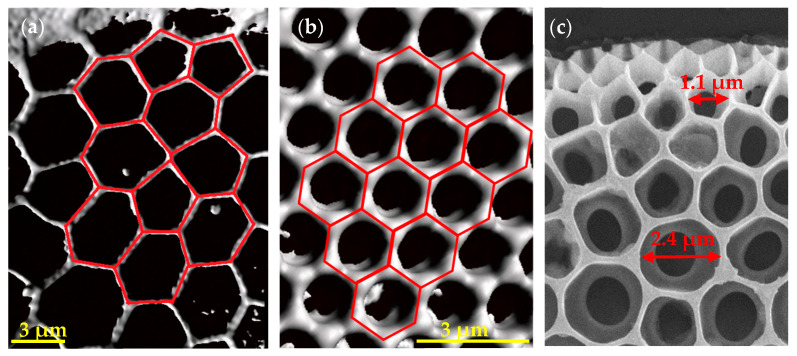
Detailed images of the pores of (**a**) *Stephanodiscus* sp., (**b**) *Thalassiosira* sp., and (**c**) likely *Porosira* sp., partly dissolved. (**a**,**b**) are images of 3D models made using CLSM. The pattern of the combs is indicated in red color; (**c**) is an SEM image.

The morphological analysis of the valves revealed that comb-like structures are mainly found in centric diatoms, and strut-like structures were mostly present in the elongated pennate diatoms. The areolae that were observed in centric diatoms often displayed polygonal combs with different characteristics. In some of the analyzed species, such as *Stephanidiscus* sp. and *Porosira* sp., an irregular pattern of polygons with a varying number of vertices was observed (Figure 9). The foramen in many centric diatoms, such as visualized in *Porosira* sp. (Figure 9c), gives the areola a sandwich-like characteristic with a cross-section resembling a T-beam. In the displayed individual, part of the frustule likely dissolved, as the outer porous layer was not present. For example, the velum, which would normally form the wall occluding an areola opposing the foramen, is missing. Another recurring feature was a size gradient, specifically seen in the areolae of species with an irregular pattern, such as *Porosira* sp., which exhibited smaller areolae towards the margin of the valve, decreasing in size by more than 50%. Other frustules exhibited areolae forming a regular pattern of hexagons, such as those present in *Thalassiosira* sp. (Figure 9b). In most diatoms, the supporting structures were positioned on the inside of the valve, with the foramen facing the inside.

Other commonly observed features are ribs and hierarchical structures, often consisting of areolae, which may form comb-like geometries, and costae, which can form ribs and ridges, as observed in the genus *Arachnoidiscus*. In many cases, several different geometrical features were present in an individual valve, varying in size. It is also to note that geometries observed in the frustules had no hard edges and corners but smooth transitions and rounded corners between structures. The valves of some frustules were undulated either on the margins (e.g., *Terpsinoë* sp.) or the valve face (e.g., *Actinoptychus* sp.), resulting in crests and troughs. These features were disregarded for this study, as the focus was set on combs.

#### 3.3.2. Comb Studies

**Figure 10:**
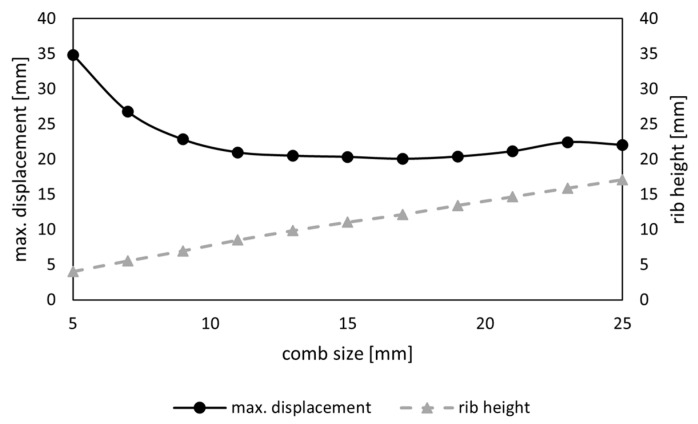
Maximum overall displacement (mm) of the model and rib height of the combs (mm) in relation to the comb size (mm).

The size study of the hexagonal comb pattern showed that $c_{s}$ is directly related to the rib height; an increase in comb size resulted in a decrease in surface area and thus enabled an increased rib height (Figure 10). The maximum overall displacement was the highest for the smallest comb size of 5 mm and gradually decreased with an increased comb size before it started to increase slightly for comb sizes larger than 17 mm. Based on these results, a comb size of 11 mm was chosen for the consecutive steps, with $\delta_{max}=20.97\;mm$. The maximum displacement was slightly lower for the comb size of 13 mm to 17 mm, but a smaller comb size was preferred to keep the overall height of the model to a minimum.

**Figure 11:**
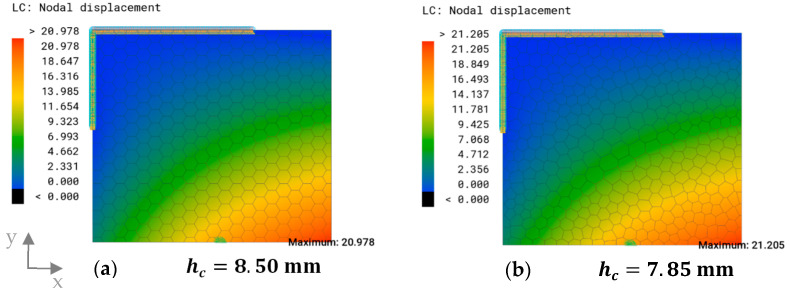
The overall displacement plot and maximum overall displacement (mm) of (**a**) the model with uniform hexagons of $c_{s}=11\;mm$ and (**b**) the model with irregular polygons with $d_{c}=11$. The rib height $h_{c}$ shows the uniform height of the comb structures.

Figure 11 illustrates the close resemblance in maximum overall displacement between the model featuring uniform hexagons and the one with irregular polygons, with a difference of less than 0.3 mm, both occurring within the same region. The height of the combs in the hexagon model was slightly higher.

**Figure 12:**
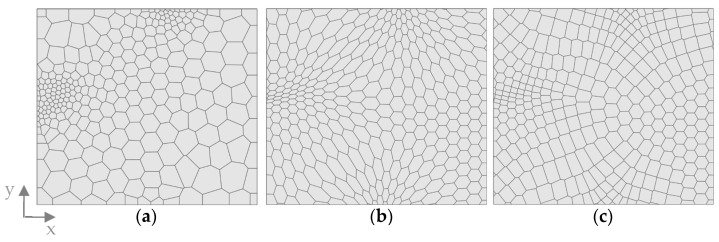
Stress-adaptive comb patterns. (**a**) pattern A: stress-adaptive randomized comb distribution; (**b**) pattern B: stress-adaptive morphed hexagon combs; (**c**) pattern C: stress-adaptive morphed hexagon center points, with subsequent Voronoi application.

All three stress-adaptive comb patterns showed a size gradient with smaller combs in the areas of high stress and larger combs in the areas of low stress (Figure 12). The combs in pattern A showed a locally restricted area of small combs, while the combs in patterns B and C depicted transformed combs in a larger area of the surface. The geometry of individual polygons varied the strongest in pattern C, with some polygons exhibiting rectangular shapes. This resulted in the formation of nearly continuous ribs that extended from the upper edge of the surface to the area of the force application.

The maximum displacement was the highest in pattern B and the lowest in pattern C, with 0.66 times the maximum displacement of the uniform hexagonal model with $c_{s}=11\;mm$ (Table 3). The rib height was the highest for pattern A and lower in patterns B and C, with almost identical values.

**Figure 13:**
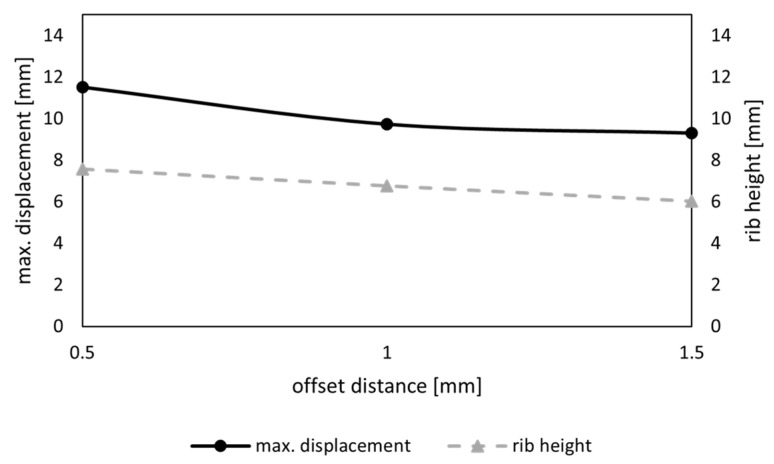
Maximum overall displacement (mm) of the model and rib height of the combs (mm) in relation to the offset distance (mm) of the sandwich surface.

The sandwich structures were applied to the model with pattern C, where an added sandwich structure decreased the maximum overall displacement of the model for all tested offset distances (Figure 13). With an increase in the sandwich offset, the maximum displacement decreased further. As the sandwich structure added mass to the model, the rib height was lower than in the previous models and decreased with a larger offset. An offset distance of $d_{o}=1.5\;mm$ resulted in an error for three of the combs that exhibited a smaller size, as the offset was too large to create valid edges. Thus, a filter was integrated into the workflow to delete invalid offsets.

#### 3.3.3. Combined Model

**Figure 14:**
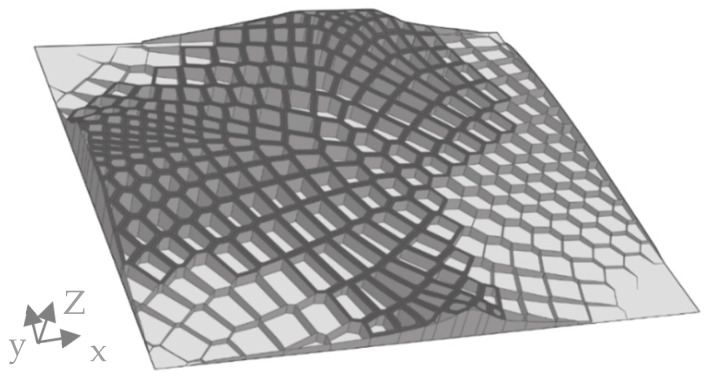
Visualization of the resulting model and its geometrical features. The dark grey areas indicate the sandwich structure, the extruded combs are colored medium grey, and the base plate is colored light grey.

The combined model consisted of pattern C of Figure 12, joined with a sandwich pattern with an offset distance of 1.5 mm, which was the largest possible offset in accordance with the comb shapes. Figure 14 shows the geometrical features of the combined model. The maximum comb height $h_{c}=15\;mm$ was distributed in the high-stress areas. The corners on the upper left and the lower right showed no combs, which is where the stress in the reference model was close to or equal to 0 MPa.

**Figure 15:**
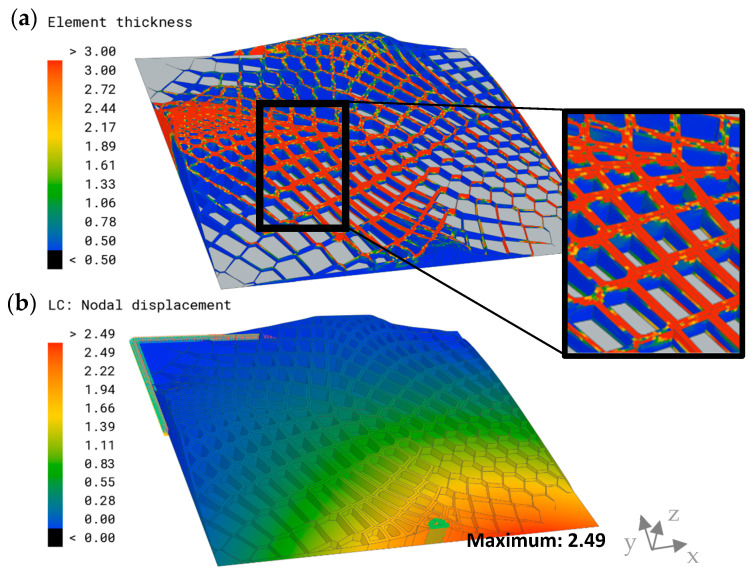
(**a**) Element thickness plot (mm) of the optimized model and a detailed view are displayed on the right. The parts excluded from the optimization are indicated in grey. (**b**) Overall displacement plot and maximum overall displacement (mm) of the combined model.

A representation of the thicknesses of the optimized model and its displacement is visualized in Figure 15. The highest thicknesses of the shell elements were observed in the sandwich structures and the upper areas of the extruded combs. The thickness decreased in the central areas of the combs, which exhibited a minimum thickness value of 0.5 mm. The displacement plot showed a similar distribution to the other models; however, it exhibited significantly lower maximum overall displacement than the models of the previous comb studies, with $\delta_{max}=2.49\;mm$.

**Figure 16:**
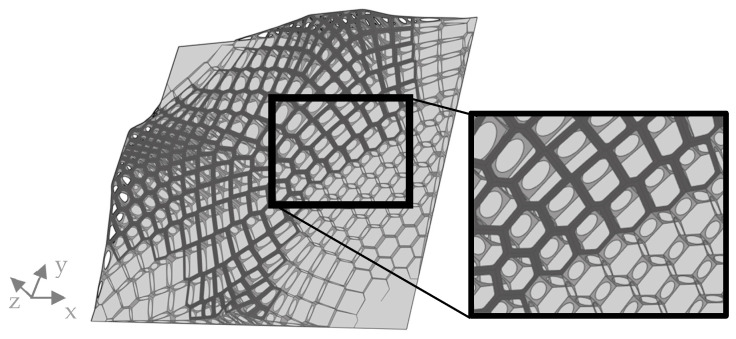
Visualization of the improved combined model, with reduced material in the combs and an increased rib height. The dark grey areas indicate the sandwich structure, the extruded combs are colored medium grey, and the base plate is colored light grey.

Based on these results, elliptical holes were cut into all comb walls, which have an area larger than 30 mm^2^ (Figure 16).

**Figure 17:**
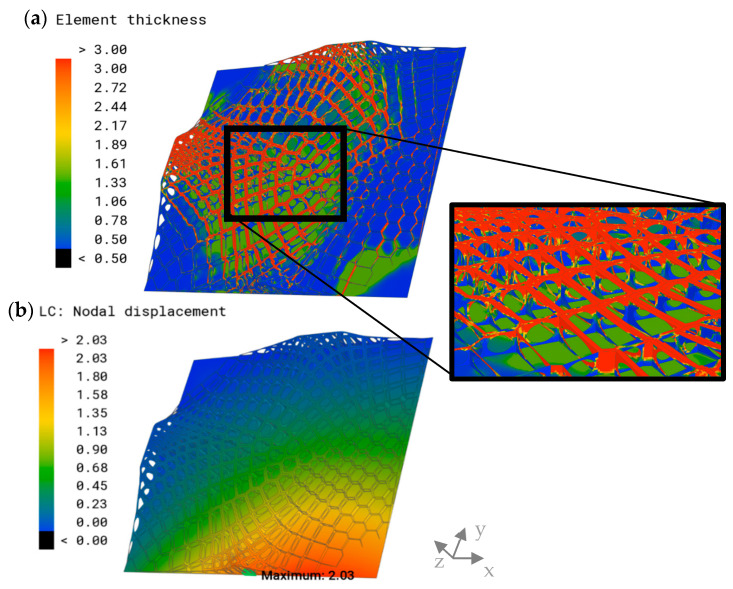
(**a**) Element thickness plot (mm) of the improved model and a detailed view are displayed on the right. (**b**) Overall displacement plot and maximum overall displacement (mm) of the improved combined model.

The distribution of element thickness and the displacement of the improved model showed similarities to the previous model (Figure 17). The addition of the plate to the thickness optimization resulted in a thickening of the plate in the central areas and in the lower right corner, where the maximum displacement was observed in most models. By applying the improvements, $\delta_{max}$ was further reduced to 2.03 mm.

### 3.4. Comparison of the Approaches

**Figure 18:**
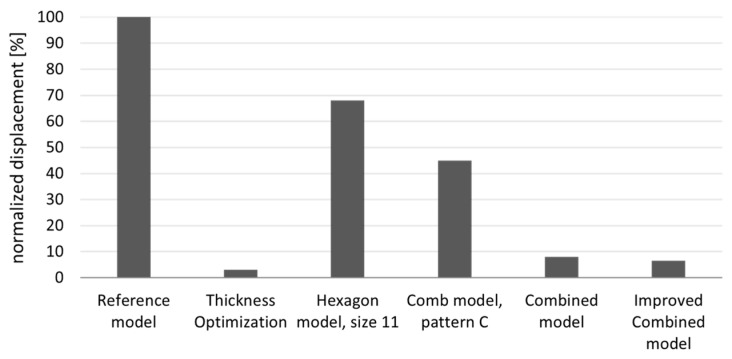
A comparative representation of the maximum displacement (%) of the key models normalized to $\delta_{max}$ of the reference model.

The maximum overall displacement of the key models, normalized to the maximum overall displacement of the reference plate, is depicted in Figure 18. Since all models had the same mass, with deviations of less than 0.1%, the displacement was comparable. All the models developed within this study exhibited better values for the target variable compared with the reference model. Among the created models, the simple uniform hexagonal model demonstrated the highest maximum displacement. Subsequent models incorporating more adjustments showed a gradual reduction in maximum displacement, with the improved combined model displaying the lowest maximum displacement among the bio-inspired models, which measured a maximum overall displacement of less than 7% of the reference model. The thickness-optimized model generally exhibited the lowest maximum overall displacement, at approximately 3% of the reference model.

### 3.5. Variation of Boundary Conditions

**Figure 19:**
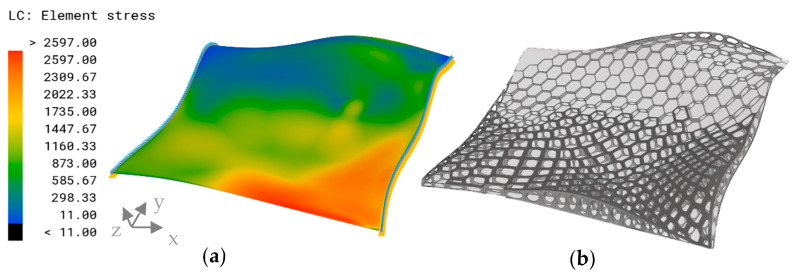
(**a**) von Mises stress distribution (MPa) in the non-planar reference surface resulting from configuration 2. (**b**) Visualization of the improved combined model approach applied to the non-planar surface. The dark grey areas indicate the sandwich structure, the extruded combs are colored medium grey, and the base plate is light grey.

The proposed approach was successfully applied to the non-planar surface. The configuration resulted in a von Mises stress distribution, as depicted in Figure 19, characterized by a prominent peak observed around the lower edge. Consequently, the generated model exhibited an enhanced rib height in this region, along with smaller deformed combs. Notably, the sandwich structure (dark grey) was observed in proximity to the same area and extended toward the center of the surface (Figure 19b). Thus, the upper edge displayed lower stress values, leading to reduced rib heights and an absence of the sandwich structure.

**Figure 20:**
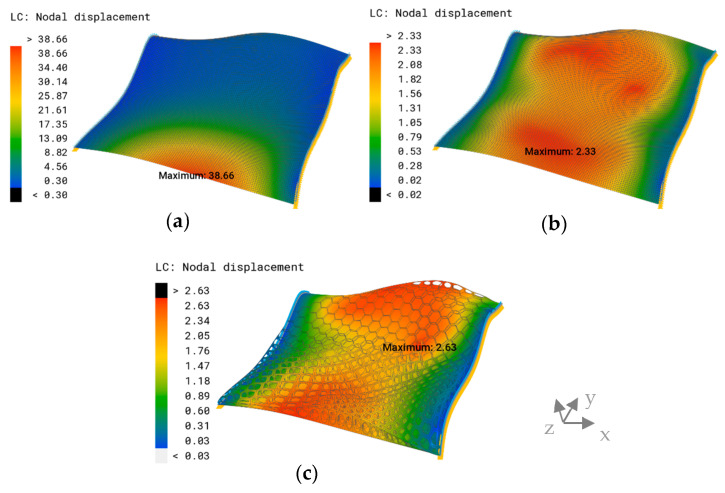
Overall displacement plot and maximum overall displacement (mm) of (**a**) the non-planar reference surface, (**b**) the thickness-optimized model, and (**c**) the improved combined model.

The non-planar reference surface with a thickness of t=2.65 mm showed a maximum displacement on the lower edge of $\delta_{max}=38.66\;mm$ (Figure 20a). The model with the implemented thickness optimization had a lower displacement of $\delta_{max}=2.33\;mm$, and the displacement was more evenly distributed throughout the model (Figure 20b). The maximum overall displacement of the combined model was $\delta_{max}=2.63\;mm$, appearing on the opposite side of the non-planar reference model (Figure 20c).

**Figure 21:**
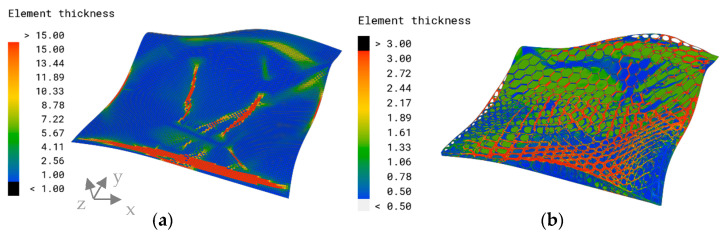
Element thickness plot (mm) of (**a**) the thickness-optimized plate and (**b**) the combined model of the non-planar plate.

The thickness optimization of the non-planar surface resulted in strut-like structures, with the most prominent one extending on the lower edge connecting the supported edges (Figure 21a). The optimized combined model exhibited a similar thickness distribution to the planar model. Greater thickness was observed in the sandwich structure and the upper parts of the comb walls (Figure 21b).

## 4. Discussion

### 4.1. Biomimetic Approach: Stress-Adaptive Sandwich Combs

#### 4.1.1. Microscopic Analysis of Diatoms

For this study, we abstracted the morphological features individually, even though they are found in complex combinations in the frustules. It is likely that the frustules of different species co-evolved into different defense strategies based on their predatory environment and the fact that the variety of feeding strategies from predators makes universal protection impossible [8]. This makes it difficult to analyze the defense strategies of the frustules as a whole; thus, the approach presented here, which examines the geometric features of the frustules individually, is more suitable and feasible.

The morphological features observed in diatom frustules, including combs, irregular and regular hexagons, rib and strut structures, and hierarchical arrangements, hold significant interest as surface stiffeners. These structural, mechanical features lead to surface stiffening, increasing the rigidity and, thus, the load-bearing capability of the diatom while using minimal material [8]. The combs, hexagons, and size gradients observed, particularly in centric diatom frustules, distribute stresses and strains effectively, enhancing overall stiffness. The walls of the areolae of many diatoms, particularly centric diatoms, form an *I*-shaped cross-section with the velum and the foramen. This shape is a favorable cross-section in terms of the second moment of area I. Compared with a simple rectangular cross-section, this results in an increased I, which simultaneously increases the bending stiffness [24].

Some observed attributes were not considered for modeling in this work. Rib and strut structures (e.g., costae), predominantly found in pennate diatoms, act as reinforcing elements, minimizing deformations and stress concentrations [7,13]. Costae surround fragile areas to deflect the stress concentrations and avoid crack propagation. Aspects such as undulations, which were observed in the valve of *Actinoptychus* sp. and the girdle region of *Terpsinoë* sp., influenced the basic shape of the valves and were thus neglected. It must be noted that these likely contribute to an increased stiffness by increasing the distance to the neutral fiber of the valve or the girdle region.

It is evident that there is a high diversity in diatom morphologies but that the individual features contributing to the mechanical performance of the frustule are similar and can be found throughout the genera in different combinations. These observed characteristics resemble several lightweight designs used in engineering solutions. I- and T-beams are commonly used, especially in architecture, for their favorable mechanical properties [2]. Additionally, sandwich-structured panels with two stiff outer layers connected by a low-strength core, which are often shaped like combs, are also employed for this reason. The remarkable diversity of diatoms, estimated to include hundreds of thousands of species [28], enables the exploration of various design principles for inclusion in the presented automated approach. Additionally, numerous publications have explored the shell structures of planktonic organisms other than diatoms, including radiolaria, tintinnids, acantharia, and foraminifera, along with potential mechanical implications [2,8,29]. Overall, morphological features of planktonic organisms provide intriguing possibilities for the design of surface stiffeners, merging efficiency, mechanical reinforcement, and lightweight architecture.

#### 4.1.2. Comb Studies

The results of the comb size and offset study both revealed that displacement is reduced by placing material further away from the neutral fiber of a structure. The reduced deflection observed in the conducted studies aligns with the previously discussed analysis of diatom frustules. The cross-section of an I-beam has a higher second moment of area than a rectangular cross-section or a T-beam [24]; thus, the models with a sandwich structure had a lower deflection than the models without. The more the material is located at the maximum possible distance from the centroid of the cross-section, such as in the form of a sandwich structure and increased rib heights, the larger the second moment of area and the higher the bending stiffness. The abstracted principles related to the increase of the second moment of the area have proven to be reliable in enhancing the stiffness of a surface.

The non-significant difference in $\delta_{max}$ of the two models with an irregular polygon pattern and the regular hexagonal pattern is likely due to the applied load case, which results in compression and tensile stress. As mentioned before, the comb walls’ contribution to the load bearing is thought to be less important. The comb pattern will likely have a more significant influence on other load cases, such as in-plane loading, which creates shear stress, which is assumed to be carried by the comb structures. Based on previous studies, it is expected that a comb pattern with high regularity results in a more even strain distribution [30,31].

The developed adaptive patterns further decreased the displacement due to a more particular material distribution, which better dissipated the emerging stress peaks through an accumulation of material in critical areas. Pattern C might have had the lowest maximum overall displacement because the performed modification of the pattern resulted in almost continuous lines of the comb walls, which expand from the support areas to the load-bearing area. The orientation of the major comb axes is thus aligned with the probable load paths. Tung et al. found that this orientation enhances the mechanical properties of a comb pattern, resulting in lower displacement [32].

#### 4.1.3. Combined Model

Evidently, the individual features made significant contributions toward reducing displacement. However, the greatest efficiency was achieved through their combined implementation in the form of the combined model. Moreover, the exploration of thickness optimization on the combined model yielded a further decrease in maximum overall displacement. The implementation of thickness optimization aligns with the intrinsic attributes of natural structures, such as those found in diatom frustules, where non-uniform thicknesses are observed [1], leading to optimized material arrangements. This optimization strategy also contributes to the reinforcement of uniform stress distribution by strategically reducing thickness in regions marked by low-stress values. Thus, the structure is more resilient towards failure [33].

It became evident that the minimal thickness allocation of the improved combined model appeared in the comb walls. This revelation underscores their limited significance in bearing loads during a bending load scenario. Notably, this observation echoes the findings of Aitken et al., who found that walls in certain diatom species primarily function to deflect shear forces rather than withstand bending loads [9]. This insight implies that the comb walls serve to maintain separation between the base layer and the sandwich construction, while their importance might become more pronounced in other load cases, such as in-plane scenarios. Given that Coscinodiscus frustules often encounter compressive and tensile forces around the girdle region, the comb walls may play an important role in mitigating these stresses [9]. Thus, the improved combined approach involving cutouts could potentially lead to increased displacement during in-plane load cases, as the structural significance of the comb walls might intensify under such conditions.

### 4.2. Comparison of the Results

Overall, the inclusion of ribbing structures, which resemble those found in diatoms, consistently outperformed the simple strategy of uniformly thickening the reference plate. This observation aligns with the findings by Hamm et al., who showed that thickened frustules with removed ribbing structures had a 70% higher displacement than ribbed models with the same total mass [4].

Notably, the engineering approach proved effective, outperforming the other approaches for the first load case. As thickness optimization is directly optimized to fit the given load case, it surpassed the efficacy of the biomimetic solutions. This outcome leaves the question of why a straightforward, thickness-optimized material distribution is not commonly observed in diatom frustules. While this principle is present in certain natural structures, such as trees or bones, which routinely encounter consistent forces, most living organisms are generalists and must be resilient toward a variety of different load cases. Diatoms serve as a prime example, as they need to adeptly manage an array of stressors introduced by various predators [8,34].

In many cases, technical problems face a very specific set of forces, such as those presented in this study, which diverge from the complex and diverse forces encountered in real-world scenarios. Thus, thickness optimization may be more applicable for specific and consistent load cases, as it is specifically adjusted to that set of forces. However, the effectiveness of thickness optimization declines when confronted with different force dynamics. The combined sandwich approach, as demonstrated, emerges as a highly promising solution with room for further improvement. Thus, it can be hypothesized that the presented biomimetic surface stiffeners are more suitable for complex load cases, as they are a more robust and versatile design, exhibiting resilience across a range of load cases. Further studies with randomized varying forces are necessary to validate this assumption.

When comparing the labor and computational effort of these approaches, the improved combined model was the most time consuming, both in creating the workflow and in terms of computational effort. The process of thickness optimization takes the most computational time, especially in the combined models, as the structures are more complex than in the reference plate. The computational time required for the improved combined model is notable, as the algorithm applied to this complex structure demands several hours for completion. Despite the initial establishment of the workflow being time consuming, its subsequent application to novel models or boundary conditions is fast and uncomplicated, requiring a few minutes for adjustments. Additionally, the combined model is set up in only one software and runs automatically after inputting the boundary conditions.

### 4.3. Variation of Boundary Conditions

The alternative configuration showed a decreased difference in maximum overall displacement for the combined model and the thickness optimization. This could underline the previously introduced hypothesis that the biomimetic approach performs better for load cases with more complex conditions and several load cases. The thickness optimization for load case 1 was specialized for one applied force; thus, it was possible to accumulate a lot of material in one area, making the model perfectly adapted to this specific load bearing. When more complex load cases are apparent, where stress concentrations are present in several different areas of the structure, simple thickness optimization might not be sufficient. The material would have to be evenly spread, resulting in a lower possible thickness due to the accumulation of a high amount of material. On the contrary, the diatom-inspired approach enables the distribution of support structures with a lower amount of material than a simple thickening. This results in a greater possible height for the structures. As the material has a bigger distance to the neutral fiber of the structure, the second moment of area is higher and, thus, increases the stiffness.

The workflow of the improved combined model was robust against the input of a modified load case but had to be adjusted to be applicable to non-planar surfaces. This included the implementation of a section of the workflow that creates a planar version of the non-planar surface before continuing with the workflow described for the planar surface. In the final section of the workflow, the created structures were morphed onto the original non-planar surface. To make the workflow applicable to both planar and non-planar surfaces at the same time, a toggle was integrated as a starting parameter, allowing the user to define whether the input surface is planar or not. Thus, the altered workflow is robust against the application to other boundary conditions. As no part of the workflow is dependent on the shape of the input surface, it is expected that the workflow is also applicable to non-rectangular surfaces.

Ultimately, this study highlighted the potential of diatom-inspired structures to increase the stiffness of surfaces. Exemplary applications include large panels, such as in aerospace engineering, as these structures could enhance durability while maintaining a lightweight design. Adaptive stiffening structures could be applied in medical devices, such as prosthetics, where high robustness is required. The parametric design would enable a fast way of creating a personalized design optimally adapted to a patient’s physical characteristics. While certain technical applications may favor conventional methods, diatom-inspired stress-adaptive designs show promise for scenarios subjected to varying stresses, necessitating lightweight and robust solutions.

## 5. Conclusions

This study aimed to evaluate the viability of the presented stress-adaptive approach as a promising alternative for addressing engineering challenges requiring increased stiffness. The implementation of the adaptive diatom-inspired approach demonstrated a significant enhancement in bending stiffness, resulting in a remarkable 93% reduction in displacement compared with the reference model with an equivalent total mass. The immediate performance of the engineering approach, exemplified in this study by simple thickness optimization, mechanically outperformed the biomimetic approach. However, it is anticipated that the inherent strength of the biomimetic approach will manifest more prominently in complex or variable load cases, which call for robust solutions, and that the biomimetic methodology will likely surpass the performance of traditional thickness optimization techniques.

Notably, the biomimetic approach achieved a high degree of automation through parametric design, resulting in the creation of a versatile workflow applicable to both planar and non-planar surfaces. This marks an initial step in automating the use of biological principles for technical applications, adapting them directly to given load cases. The significant diversity among plankton organisms implies the potential to extend additional biological principles into automated technical tools through the demonstrated approach.

## Figures and Tables

**Table 1:** Material properties of structural steel, which was used for the simulation of all models.

Property	Value
Density [kg m^−3^]	8000
Young’s modulus [MPa]	193,000
Poisson ratio [-]	0.28
Maximum yield stress [MPa]	360

**Table 2:** Stress intervals in the percentage of the maximum stress threshold of 600 MPa and the assigned structural features for each interval.

$\sigma_{VM}$ [%]	Comb Height (mm)	Sandwich Structure
<10%	0	no
10–40%	1–15	no
40–100%	1–15	yes

**Table 3:** Maximum overall displacement $\delta_{max}$ (mm), maximum displacement normalized to $\delta_{max}$ of the hexagonal model with $c_{s}=11\;mm$ (see Figure 11a) and the rib height $h_{c}$ (mm) of the three patterns.

Pattern	$\delta_{max}$ (mm)	$\delta_{max}$ $/\delta_{max,hexagon}$	$h_{c}$ (mm)
A	14.80	0.71	10.30
B	19.51	0.93	8.40
C	13.87	0.66	8.48

## Data Availability

The data presented in this study are available on request from the first author.
